# Impact of yam bean pulp on growth performance, gut morphology, digestive development, and digestibility in broilers raised in hot environments

**DOI:** 10.5713/ab.25.0057

**Published:** 2025-05-19

**Authors:** Kedsirin Sakwiwatkul, Wannisa Ojan, Purinut Treehera, Jessada Pidtathasa, Theeranon Pomwong, Anut Chantiratikul, Siriporn Lawan, Manisa Sangkaew

**Affiliations:** 1 Major of Animal Science, Department of Agricultural Technology, Faculty of Technology, Mahasarakham University, Maha Sarakham, Thailand; 2 Department of Food Technology, Faculty of Technology, Mahasarakham University, Maha Sarakham, Thailand

**Keywords:** Broilers, Dietary Fiber, Digestive Traits, Histomorphology, Hot Environment, Nutrient Digestibility

## Abstract

**Objective:**

Rearing broilers in high-temperature environments can impair growth performance, affecting the economic efficiency of broiler production. Dietary fiber sources like yam bean pulp (YBP) may mitigate these effects by promoting digestive organ development and gut integrity, enhancing nutrient absorption and growth. This study aimed to evaluate the impact of YBP inclusion on digestive development, gut morphology, nutrient digestibility, serum lipid profiles, and growth performance in broilers raised under high environmental temperatures.

**Methods:**

Since YBP has not previously been used in poultry diets, its optimal inclusion level was determined using *in vitro* digestibility measurements, which identified 4% YBP as the optimal initial inclusion level for further *in vivo* study. Subsequently, the *in vivo* study was conducted with 200 one-day-old ROSS broiler chicks, randomly assigned to four dietary treatments (0%, 4%, 8%, and 12% YBP; 5 replicates of 10 birds each), and housed in an open system at an average temperature of 32.1±3.7°C. Digestive traits, nutrient digestibility, serum lipid profiles, and growth performance were assessed over 21 days.

**Results:**

Results showed that YBP inclusion (up to 12%) improved gut morphology by increasing villi height and the villi height-to-crypt depth ratio in the duodenum and ileum, alongside dose-dependent improvements in dry matter digestibility. However, crude protein digestibility declined when YBP levels exceeded 8%. Despite these benefits, YBP inclusion did not alleviate heat stress effects on growth performance, digestive organ development, or serum lipid profiles, even at the highest inclusion level.

**Conclusion:**

Dietary YBP can enhance gut morphology and nutrient digestibility in broilers from day 1 to 21, with optimal inclusion levels not exceeding 8%. However, its use does not mitigate the negative effects of heat stress on broiler growth in high-temperature environments.

## INTRODUCTION

High environmental temperatures negatively impact poultry health and productivity, leading to heat stress and economic losses, particularly in Southeast Asian’s broiler farms, such as those in Thailand. Heat stress can negatively affect the overall performance of chickens and weaken their immunity, potentially leading to higher mortality [1]. To address this issue, using dietary fiber as a functional feed additive to enhance growth performance during heat stress is one of the strategies. As is known, dietary fiber supports gastrointestinal integrity and function by increasing villi height and hepatic enzyme activity, which enhances nutrient digestion and absorption [2,3]. This improvement aids early digestive development in chickens and later boosts their performance. Moreover, dietary fiber intake has been observed to enhance the immune system by increasing immune organ weight, boosting gene expression, and promoting antibody production [4], potentially counteracting weakened immunity caused by heat stress. Given these benefits, early fiber intake is speculated to help mitigate the effects of heat stress on broiler performance in hot climates. Regarding the above, yam bean fiber is a promising candidate, as it demonstrates significant potential due to its effectiveness against various diseases and its well-recognized medicinal properties [5,6], including antioxidant effects [7,8].

Yam bean pulp (YBP), a fiber-rich by-product of yam bean (*Pachyrhizus erosus*) juice processing, stands out as an intriguing candidate as a promising feed additive. Abundantly grown in Mahasarakham province, Thailand, yam bean tubers are known for their high fiber content and bioactive compounds, such as inulin, flavonoids, and phenolics [9]. Notably, it contains low levels of antinutritional components [10], highlighting the potential of the *P. erosus* tuber as a valuable feedstuff. Various studies have demonstrated the health benefits of yam bean fibers, including reducing oxidative stress by preventing malondialdehyde increase in mice fed a high-fat diet [7], enhancing immunity by stimulating proinflammatory cytokines TNF‐α and IL‐6 while suppressing IL‐10 [11], and promoting the abundance of beneficial microbial species while inhibiting pathogenic ones in the gut [12]. Despite the reported positive effects of yam bean fiber, most studies have been conducted on mice, with no evidence of its impact on gut morphology modulation. Therefore, research on the use of YBP as a poultry feed ingredient remains limited.

Due to the limited application of YBP in poultry feed, this study conducted an *in vitro* digestibility analysis to determine the optimal inclusion level of YBP in broiler diets without compromising nutrient digestibility. This was followed by an *in vivo* study to assess the effects of YBP inclusion on digestive organ development, gut morphology, nutrient digestibility, and growth performance in broilers raised in hot environments. Additionally, serum lipid profiles were measured, as yam beans may have lipid-lowering effects in the blood, potentially due to their high fiber [8] and inulin content [13].

## MATERIALS AND METHODS

### Experiment 1: *in vitro* study

#### YBP preparation and chemical analysis

Yam bean (*P. erosus*) tubers, harvested at 3 months of growth, were commercially obtained from local farms in Borabue District, Maha Sarakham Province, Thailand (latitude 16°2′18″N, longitude 103°7′9″E). The tubers were thoroughly washed with tap water, peeled, finely chopped, and then juiced to extract fresh YBP. Subsequently, the fresh YBP was dried in a hot air oven (Memmert Universal Oven UF 450; Memmert, Schwabach, Germany) at 50°C until the moisture content was reduced to less than 10%. After drying, the YBP was ground to pass through a 1.0-mm mesh screen. It was then stored at room temperature (approximately 28.7±1.4°C) for further chemical analysis, including proximate compositions [14], inulin content [15], total phenolic acid [16], total flavonoids [17], 2,2-diphenyl-1-picrylhydrazyl assay (DPPH) [18], and ferric reducing/antioxidant power (FRAP) [19], as detailed in Table 1.

#### Dietary treatments and in vitro digestibility assay

To determine the optimal inclusion level of YBP in the poultry diet, six diets were formulated with YBP levels of 0%, 2%, 4%, 6%, 8%, and 10% (Table 2), substituting maize. Diets were formulated to meet the nutrient requirements for the broiler starter phase as recommended by NRC [20]. The *in vitro* digestibility of the diets was evaluated using methods adapted from Saunders et al [21], to align with poultry body conditions. Briefly, each diet sample (approximately 250 mg) was suspended in 15 mL of 0.1 N HCl with pepsin (>10,000 NFU/mg, HiMedia Laboratories, Thane, India) at 41°C for 3 hours. After neutralization with 0.5 N NaOH, the mixture was combined with phosphate buffer (pH 8.0) containing pancreatin (>100 USP U/mg amylase, >100 USP U/mg protease, >8 USP U/mg lipase, HiMedia Laboratories) and then shaken at 41°C for 24 hours. The mixture was subsequently filtered, rinsed with distilled water, and then dried. Dry matter (DM) and crude protein (CP) analyses of the diet and dried digesta were used to calculate the *in vitro* digestibility of DM (IVDMD) and CP (IVCPD).

### Experiment 2: *in vivo* study

#### Dietary treatments and husbandry

Four dietary treatments were tested: a control group without YBP and three groups in which YBP was included at levels of 4%, 8%, and 12%, replacing maize in the diets, based on the *in vitro* study (Table 3). All diets were formulated to be isocaloric and isonitrogenous, meeting or slightly exceeding the nutrient requirements specified by the ROSS broiler nutrition guidelines [22]. In the last three days of the experimental period, titanium dioxide (TiO_2_; Sigma-Aldrich, St. Louis, MO, USA) was added at 0.5% as an indigestible marker for ileal digestibility measurement. The experiment was conducted from March 15 to April 4, 2024, during the summer season in Thailand. A total of 200 birds, one-day-old ROSS broiler chickens, were randomly allocated to four dietary treatments in a completely randomized design, with each treatment consisting of 5 replicates of 10 birds each. Birds were housed in a conventional open housing system with an average temperature of 32.1±3.7°C during the 21-day rearing period. They were exposed to high daytime temperatures for approximately 12 hours, followed by a period of thermal relief during the night. Birds were kept in litter-floor pens (1.5×2 m) following the lighting program recommended by the ROSS broiler management. Feed and water were provided *ad libitum*.

#### Experimental procedure and sample analysis

During the trial, the birds’ body weight (BW) was measured on the initial day and then weekly, along with their daily feed intake (FI). These data were used to calculate BW gain, average daily gain (ADG), and feed conversion ratio (FCR), in which FCR is calculated as total FI divided by total weight gain.

On the last of the experimental period, after fasting, two birds from each replicate (10 birds per treatment) were randomly selected for the blood collection. Blood samples were drawn from the jugular vein and allowed to clot in polypropylene tubes at room temperature for collecting blood serum. The serum was then used for the analysis of triglycerides, cholesterol, and low-density lipoprotein (LDL) using a spectrophotometric method with an OLYMPUS AU400 Chemistry Analyzer (Beckman Coulter, Brea, CA, USA). Subsequently, three birds from each replicate (15 birds each treatment) were euthanized by cervical dislocation, and the digestive organs were eviscerated to assess digestive organ traits and gut histomorphology. To evaluate the digestive organ traits, the proventriculus, gizzard, liver+gallbladder, duodenum, jejunum, and ileum were weighed, and their relative weights were expressed as g/100 g of live BW. The lengths of small intestine segments were measured and expressed as cm/100 g of live BW.

#### Gut histomorphology measurement

The small intestinal sections were prepared and measured the villus height (Vh), crypt depth (Cd), and the ratio between them (Vh:Cd ratio), following the method described by Sabour et al [23]. Approximately 2 cm long segments were collected from the duodenum, jejunum, and ileum, and flushed gently with physiological saline solution (0.9% NaCl) to remove intestinal contents. Subsequently, the segments were stored in 10% formalin solution for fixation. The samples were processed for 24 hours in a tissue processor using ethanol as the dehydrating agent and subsequently embedded in paraffin. Sections of 5 μm thickness were prepared using a semi-automated rotary microtome (Model RM2245; Leica Biosystems, Nussloch, Germany) and stained with hematoxylin-eosin. Intestinal morphology was examined using a compound light microscope (Eclipse E200; Nikon, Melville, NY, USA) with an EOS-600D Canon camera. A total of 10 intact, well-oriented villus-crypt units were selected from each intestinal cross-section, with three cross-sections per sample. Vh (in micrometers) was measured from the tip of the villus to the villus-crypt junction, while Cd was defined as the depth of the invagination between two villi. Subsequently, the Vh:Cd ratio was calculated.

#### Digestibility measurement

On day 22, after fasting, the birds were fed experimental diets for 4 hours before euthanasia. Subsequently, ileal digesta were collected (5 birds per replicate) from the distal two-thirds of the ileum, extending from Meckel’s diverticulum to approximately one inch anterior to the ileocecal junction. The digesta samples were dried and ground to pass through a 1.0-mm mesh sieve for DM and CP analysis following the standard method of AOAC [14]. The TiO_2_ concentrations in the diets and ileal digesta were measured using the method described by Short et al [24]. These TiO_2_ concentrations, along with nutrient levels in both the diets and ileal digesta, were used to calculate digestibility. The apparent ileal digestibility (AID) of nutrients was calculated using the following Eq. 1 [25]:


(1) $$\text{AID of nutrient }(\%)=[\{{\left(\text{nutrient}/\text{Ti}\right)}_{\text{d}}-{\left(\text{nutrient}/\text{Ti}\right)}_{\text{i}}\}/{\left(\text{nutrient}/\text{Ti}\right)}_{\text{d}}]\times 100$$

Where (nutrient/Ti)_d_ represents the ratio of nutrient to Ti in the diet, and (nutrient/Ti)_i_ represents the ratio of nutrient to Ti in the ileal digesta.

### Statistical analysis

The data were analyzed using one-way analysis of variance. In instances where significant differences were found, means were assessed separately using the Tukey-Kramer test. All statistical procedures were conducted with the SAS University Edition [26]. Statistical significance for all hypothesis tests was determined at p<0.05.

## RESULTS

### Experiment 1: *in vitro* study

#### Chemical compositions in yam bean pulp

The nutritional composition of YBP primarily consisted of 17.2% fiber and 65.6% nitrogen-free extract (NFE), with minor amounts of protein and ash, each around 8%. It contained less than 1% fat (Table 1). Additionally, YBP was found to contain approximately 10.7% inulin and high levels of antioxidant compounds, including 664.2 μg/g of total flavonoids and 438.2 μg/g of total phenolic acids. Moreover, YBP demonstrated high antioxidant activity, as measured by DPPH and FRAP assays, and had digestibility values of approximately 56% IVDMD and 54% IVCPD.

#### In vitro nutrient digestibility

**Figure 1:**
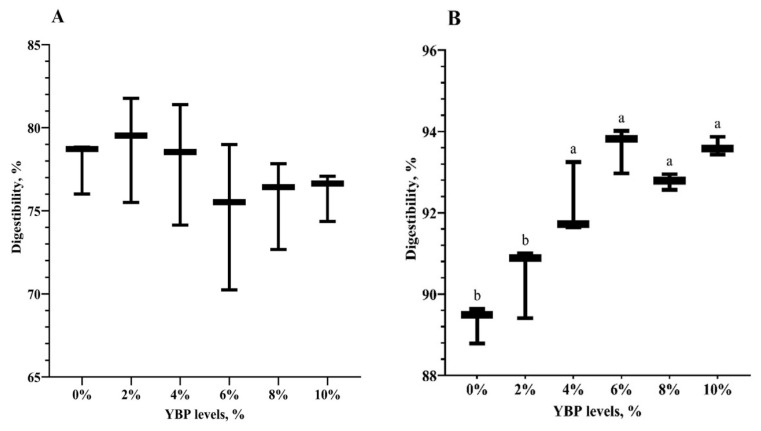
Effects of inclusion levels of yam bean pulp (YBP) on *in vitro* digestibility of dry matter (A) and crude protein (B). The diets included varying amounts of YBP at 0%, 2%, 4%, 6%, 8%, and 10%. Data represent mean±standard deviation (n = 3). ^a,b^ Different superscript letters indicate significant differences at the p<0.05 level. YBP, yam bean pulp.

The IVDMD of the 0% YBP diet measured 77.9% and remained unchanged with YBP additions up to 10% (Figure 1). In contrast, the IVCPD was significantly influenced by increasing YBP levels. The diet without YBP had the IVCPD of 89.3%, which increased in a dose-dependent manner with higher YBP levels. Notably, the IVCPD at 4% YBP was significantly higher than the 0% YBP group but did not differ significantly from that at 6%, 8%, and 10% YBP levels.

### Experiment 2: *in vivo* study

#### Digestive organ traits and gut histomorphology

**Figure 2:**
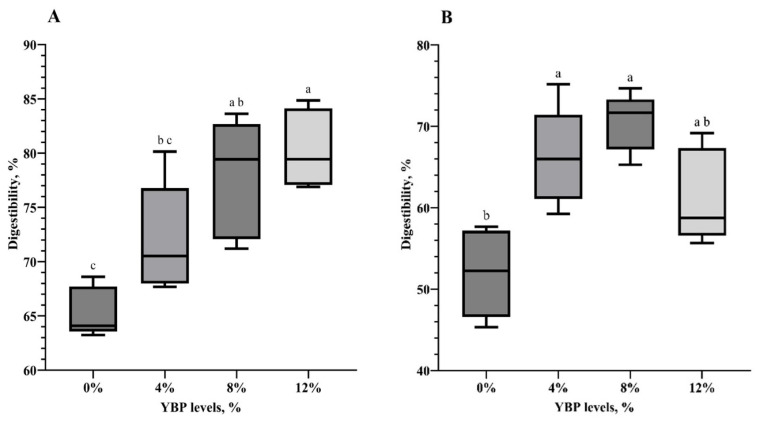
Effects of inclusion levels of yam bean pulp (YBP) on the apparent ileal digestibility of dry matter (A) and crude protein (B) in broilers rearing to 21 d of age. The diets included varying amounts of YBP at 0%, 4%, 8%, and 12%. Data represent the mean±standard deviation of 5 replicate pens per treatment with 5 birds per replicate. ^a−c^ Different superscript letters are significantly different at the p<0.05 level. YBP, yam bean pulp.

**Figure 3:**
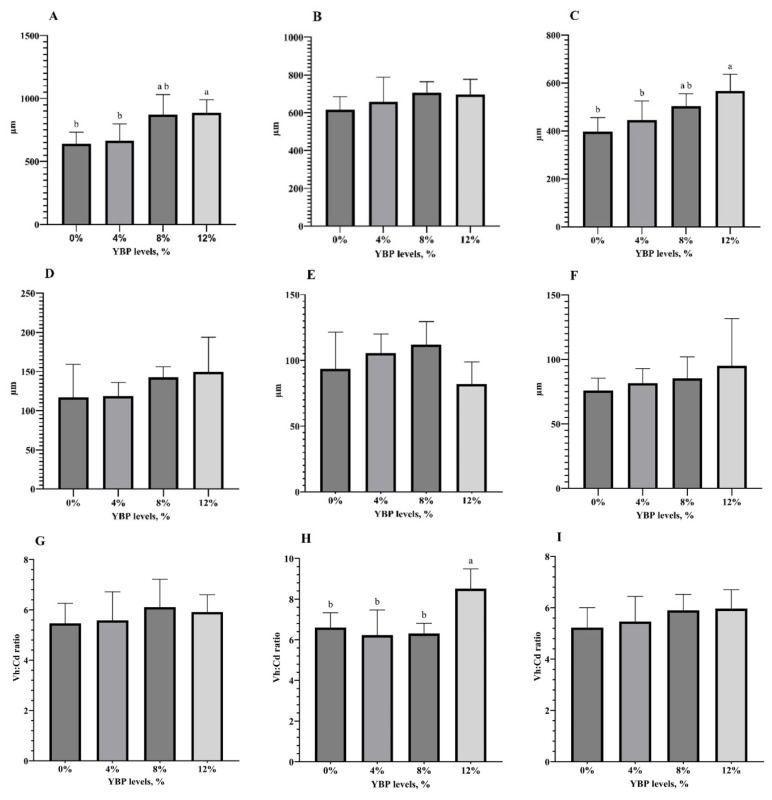
Effects of yam bean pulp (YBP) inclusion in diets on small intestine histology in broilers up to 21 days of age. Diets contained YBP at 0%, 4%, 8%, and 12%. Measurements included villus height in the duodenum (A), jejunum (B), and ileum (C); crypt depth in the duodenum (D), jejunum (E), and ileum (F); and the villus height-to-crypt depth ratio (Vh:Cd ratio) in the duodenum (G), jejunum (H), and ileum (I). Data are presented as mean ± standard deviation from five replicate pens per treatment, with three birds per replicate. ^a,b^ Means with different superscript letters differ significantly at p<0.05. YBP, yam bean pulp.

The effects of dietary YBP on promoting the development of digestive organs are shown in Table 4. The weights of the proventriculus, gizzard, liver+gallbladder, and sections of the small intestines did not differ significantly between groups fed diets with added YBP and those with 0% YBP. However, increasing YBP levels led to higher Vhs in the duodenum and ileum (p<0.05), though not in the jejunum (Figures 2, 3). YBP inclusion did not alter the Cd or Vh:Cd ratio in any section of the small intestine, except for the jejunum, where it significantly affected the Vh:Cd ratio. Birds fed 12% YBP exhibited the highest Vh:Cd ratio among the groups (p<0.05).

#### The apparent ileal digestibility

**Figure 4:**
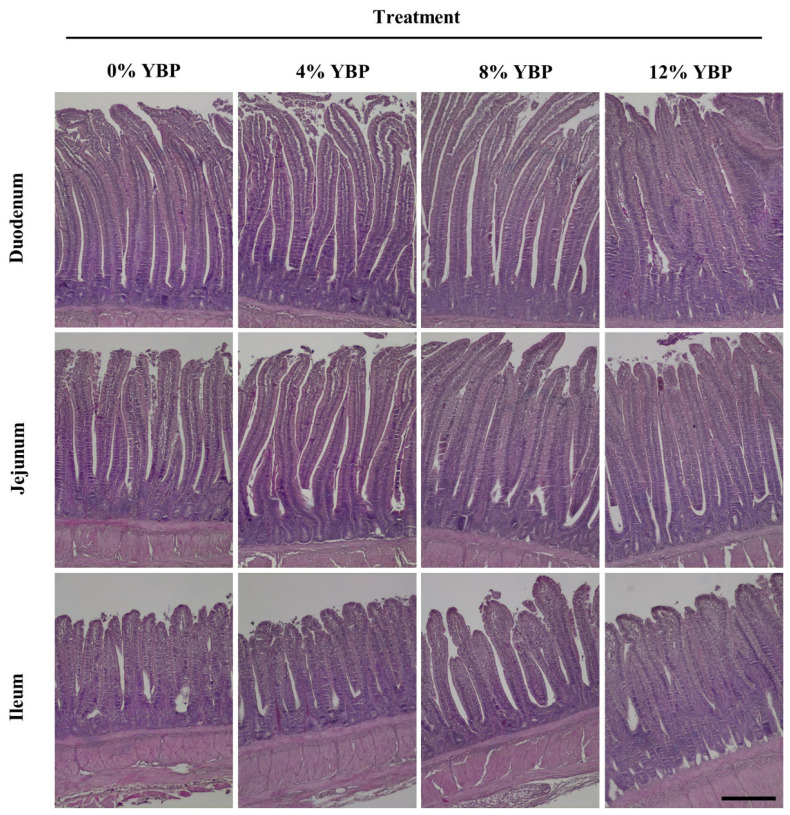
Effect of including yam bean pulp (YBP) in diets on the histology of small intestine in broilers reared up to 21 days of age. Tissues of duodenum, jejunum and ileum were sectioned and stained by H&E dye. Tissues of the duodenum, jejunum and ileum were sectioned and stained by Hematoxylin and Eosin stain. Treatments consisted of diets with 0% YBP, 4% YBP, 8% YBP, and 12% YBP. Scale bar = 100 μm. YBP, yam bean pulp.

Ileal DM digestibility was around 65% in the group fed with 0% YBP, and this value increased gradually in those fed with higher levels of YBP (p<0.05), as illustrated in Figure 4. The DM digestibility increased from approximately 65% in the 0% YBP group to a peak of 80.4% with 12% YBP inclusion (p<0.05). A similar trend was observed in the digestibility of CP, which increased from around 52.0% in the group that was fed a 0% YBP diet to a peak of 70.5% with 8% YBP inclusion (p<0.05). There were no significant differences in CP digestibility among the YBP-added groups. However, YBP inclusion levels above 8% tended to reduce CP digestibility; specifically, inclusion of 12% YBP resulted in approximately a 13.1% decrease in CP digestibility.

#### Serum lipid profiles and growth performance

The results indicated that varying levels of YBP inclusion in the diets did not influence the modulation of serum lipid profiles, including triglycerides, cholesterol, and LDL levels (Table 5).

No significant differences were observed among treatments in terms of growth performance parameters (Table 6). However, it was noticed that higher levels of YBP tended to improve the performance of broiler chickens at an early age. Increasing levels of YBP in the diets appear to be associated with higher BW gain, ADG, and improved FCR in a dose-dependent manner.

## DISCUSSION

### Experiment 1: *in vitro* study

Research on the chemical composition and digestibility of YBP remains limited; therefore, this study aims to provide important information on its chemical properties and potential as a poultry feed ingredient. YBP was primarily composed of NFE (66%) and fiber (17%). High NFE in YBP may serve as an energy source for poultry feed, while the fiber supports digestive organ development and improves gut morphology in chickens. It also contained approximately 11% inulin, which can benefit poultry gut health by acting as a prebiotic, promoting the growth of beneficial bacteria, and inhibiting pathogens, as reported by Xia et al [27]. In addition, YBP contained high levels of total flavonoids and phenolics, with strong antioxidant activity demonstrated through FRAP and DPPH assays, suggesting its potential as a feed additive to mitigate the negative effects of rearing broilers in hot climates. The *in vitro* digestibility of YBP was assessed, showing IVDMD at 56% and IVCPD at 54%, both lower than maize, which has DM digestibility of 65%–70% [28] and CP digestibility of 79%–82% [29] in chickens. Based on the above, YBP’s high fiber and phytochemical content make it a potentially beneficial feed additive for poultry. However, its low digestibility raises concerns, so careful consideration of the appropriate YBP level is necessary.

As YBP has not been previously used in poultry diets, this study aimed to determine its optimal inclusion levels for broiler feed. Including YBP up to 10% did not significantly affect IVDMD, suggesting it can be used safely as a high-level ingredient without adversely impacting nutrient digestibility. On the other hand, although all diets were isonitrogenous, higher YBP levels improved IVCPD, possibly due to the presence of proteolytic enzymes in YBP. While amylolytic enzymes in yam bean (*P. erosus*) tubers have been reported by Noman et al [10], there is currently no information on the presence of proteolytic enzymes in yam beans, making this a worthwhile area for future research. In addition, although IVCPD increased with higher YBP levels, no significant differences were observed among diets containing 4% YBP or higher. Therefore, including 4% YBP in broiler diets could serve as a suitable starting point for further *in vivo* studies.

### Experiment 2: *in vivo* study

Studies have shown the beneficial effects of dietary fiber on digestive organ development by stimulating the muscular activity of the proventriculus and gizzard, and increasing the functional activity of other related organs, resulting in larger sizes and heavier weights, as reported by Tejeda and Kim [3] and González-Alvarado et al [30]. This improvement is especially notable with intake of insoluble fiber, as its larger particles prolong gizzard retention time until reaching a minimal critical size [30]. Additionally, insoluble fiber stimulates the secretion of pancreatic enzymes (e.g., amylase, lipase, proteases), thereby improving nutrient digestibility [3]. However, this study found that YBP inclusion, reaching 12% (4.9% fiber in the diet, 1.5 times higher than the 0% YBP diet), did not affect the development of digestive organs. It is well established that insoluble fiber significantly impacts digestive organ size, while soluble fiber has a lesser effect. This may explain the current findings, suggesting that YBP fiber is likely soluble. However, the fiber type in YBP was not determined, indicating the need for further analysis.

Structural features of the small intestine, such as Vh, Cd, and the Vh:Cd ratio, indicate segment-specific digestive roles and absorptive efficiency. Vh is positively associated with nutrient absorption, while crypts support epithelial renewal, with their depth reflecting tissue turnover. Although necessary, deeper crypts may also suggest increased endogenous protein loss. The Vh:Cd ratio is a key indicator of intestinal absorptive capacity [31]. Interestingly, these intestinal features can be modulated by dietary fiber. In the present study, the addition of YBP significantly improved gut morphology in the small intestine, as evidenced by increased villi height in the duodenum and ileum, along with a higher Vh:Cd ratio in the ileum. These enhancements were observed in a dose-dependent manner, with the most pronounced effects seen at a dietary inclusion level of 12% YBP. These findings aligned with previous studies [2,4], which reported that increasing fiber levels in the diet can positively influence gut integrity. Increasing gut villi height effectively promotes nutrient absorption by expanding the small intestine’s surface area [32], boosting brush border enzyme production and nutrient transporter availability [33], which enhances nutrient uptake and, consequently, growth rates in chickens.

Interestingly, the addition of YBP significantly improved nutrient digestibility. Increasing the YBP level led to a linear rise in DM digestibility, even at 12% YBP. This improvement may be associated with enhancements in gut morphology at increasing levels of YBP, as discussed earlier, particularly in the duodenum and ileum: the former is the primary site of enzyme secretion and nutrient digestion, while the latter is the main site for nutrient absorption [34].

Additionally, adding YBP up to 8% significantly enhanced CP digestibility. Since all diets were isonitrogenous, the improved CP digestibility with higher YBP levels can probably be attributed to two factors: (1) increased production of brush border enzymes and nutrient transporters due to the enhanced intestinal surface area from YBP fiber intake, and (2) the presence of proteolytic enzymes in YBP, as suggested in our previous *in vitro* study. On the other hand, the inclusion of YBP above 8% tended to affect CP digestibility negatively. This is unlikely due to the 4.9% fiber in the 12% YBP diet, as fibrous feedstuffs at 3%–5% levels do not compromise digestibility or growth in broilers from day 1 to 21, as reported by various studies [35–37]. Moreover, 7%–9% fiber inclusion could even promote performance in broilers from day 22 to 42 [4]. Indeed, the decline in CP digestibility at YBP levels exceeding 8% may be due to an unknown antinutritional factor present in YBP. Therefore, further exploration of the antinutritional factors limiting the use of YBP may be needed.

Dietary fiber could reduce the reabsorption of bile acids in the intestine, which can result in a reduction of cholesterol levels in the bloodstream [38]. This aligns with the findings of Bogusławska-Tryk et al [39] and Okrathok and Khempaka [40], who reported fiber’s beneficial effects on reducing serum cholesterol, triglycerides and LDL in chickens. Unfortunately, this effect was not observed in our study, despite increasing crude fiber to 4.9% in the diet with 12% YBP inclusion. In this context, the beneficial effect of YBP fiber on lowering serum lipids may be diminished by higher dietary fat levels required to maintain isocaloric conditions in all YBP diets, as YBP provides less metabolizable energy (594 kcal/kg, calculated) than maize (3,350 kcal/kg, NRC [20]).

The average ADG and FCR of commercial broilers at 21 days old have been reported as 62.4 g/day and 1.38, respectively, in the study by Zuidhof et al [41]. This report is in accordance with Teixeira Netto et al [42], who observed an average FCR of 1.45 and 1.49 for broilers fed mash and pellet feed, respectively, from 1 to 21 days old. These findings differ from our observations, where birds fed a 0% YBP diet had an average ADG of 15.7 g/day and an FCR of 2.18, both of which were lower than previously reported values. This decline may be attributed to hot ambient conditions and associated heat stress, which reduced average feed consumption compared to the recommended performance objectives for ROSS broilers [43], as the study was conducted in an open system with high temperatures averaging 32.1±3.7°C during the experimental period.

Additionally, the inclusion of YBP did not affect growth performance, even when YBP levels were increased to 12%. It seemed that increasing YBP levels in the diet could not mitigate the adverse effects of heat stress, despite YBP containing antioxidant components and exhibiting antioxidant activities, as shown in Table 1. This suggests that the antioxidant levels in YBP are insufficient to counteract oxidative stress in birds; therefore, higher YBP levels may be required to address this issue. In addition, although the inclusion of YBP showed beneficial effects on gut morphology and nutrient digestibility in broilers, as observed earlier, these improvements only marginally alleviated the impaired growth performance of broilers raised in a hot environment. This limited impact on growth might be explained by an increased nutrient demand required to support higher epithelial cell turnover in the gut villi, as influenced by dietary fiber inclusion [3,35], despite the observed improvement in protein digestibility. Consequently, understanding the balance between protein synthesis, protein turnover, and growth, as regulated by dietary fiber inclusion is crucial and requires further research to optimize chicken performance.

## CONCLUSION

Dietary YBP primarily comprised NFE and fiber, with minimal levels of other nutrient components. Additionally, YBP contained high concentrations of phenolics and flavonoids, exhibiting strong antioxidant capacity. The inclusion of YBP in broiler diets positively influenced gut morphology in both the anterior and distal regions of the small intestine, as well as DM digestibility, in a dose-dependent manner. However, CP digestibility improved only when YBP inclusion levels were maintained at or below 8%. Although gut morphology and nutrient digestibility improved with increasing levels of YBP inclusion, these benefits were not sufficient to counteract the negative effects of heat stress on the growth performance of chickens. Based on these findings, dietary YBP holds promise as a feed additive for enhancing gut morphology and nutrient digestibility in broiler chicks, provided its inclusion level does not exceed 8%. However, its beneficial effects were not evident under heat stress conditions.

## Figures and Tables

**Table 1:** Chemical composition, antioxidant property, and *in vitro* nutrient digestibility of yam bean pulp

Items	Yam bean pulp	Items	Yam bean pulp
Crude protein (%)	8.79±0.07	Total flavonoid (μg/g)	664.23±15.37
Ether extract (%)	0.22±0.03	Total phenolic acid (μg/g)	438.23±1.22
Crude fiber (%)	17.16±1.14	FRAP (μg/g)^1)^	618.64±3.74
Crude ash (%)	8.18±0.21	DPPH (μg/g)^1)^	134.34±2.72
Nitrogen free extract (%)	65.64±1.18	IVDMD (%)	55.52±0.55
Inulin (mg/g)	10.68±0.10	IVCPD (%)	54.05±0.14

The values of each parameter represent the mean values±SD of triplicate analyses (in dry matter).

1) The 2,2-diphenyl-1-picrylhydrazyl (DPPH), and ferric reducing/antioxidant power (FRAP) assay are used to predict antioxidant activities by inhibiting lipid oxidation.

IVDMD, *in vitro* dry matter digestibility; IVCPD, *in vitro* crude protein digestibility; SD, standard deviation.

**Table 2:** Composition and nutrient level of the experimental diets for *in vitro* experiment

Ingredients, % (as-fed basis)	Dietary groups					

	0% YBP	2% YBP	4% YBP	6% YBP	8% YBP	10% YBP
Maize	52.7	50.2	47.3	44.7	41.9	39.3
Soybean meal	41.2	41.2	41.5	41.6	41.8	41.9
YBP	0.0	2.0	4.0	6.0	8.0	10.0
Palm oil	1.6	2.1	2.6	3.2	3.7	4.2
DL-methionine	0.2	0.2	0.2	0.2	0.2	0.2
L-lysine	0.5	0.5	0.5	0.5	0.5	0.5
Vit-mineral premix	0.3	0.3	0.3	0.3	0.3	0.3
Dicalcium phosphate	2.1	2.1	2.1	2.1	2.2	2.2
Calcium carbonate	1.0	1.0	1.0	1.0	1.0	1.0
Salt	0.5	0.5	0.5	0.5	0.5	0.5
Analyzed value (%)^1)^						
Crude protein	21.1±1.01	21.3±2.71	20.7±0.36	20.7±0.15	21.7±0.94	21.5±1.20
Crude fiber	3.3±0.42	3.6±0.30	3.8±0.25	4.1±0.34	4.4±0.37	4.7±0.57

Vit-mineral premixes provided the following per kilogram of premix: vit A, 1,500,000 IU; vit D3, 300,000 IU; vit E, 2,400 IU; vit K, 300 mg; thiamin, 300 mg; riboflavin, 600 mg; pyridoxine, 300 mg; vit 12, 4 mg; pantothenic acid, 1,500 mg; folic acid, 60 mg; nicotinic acid, 2,000 mg; biotin, 10 mg; copper, 24,000 mg; manganese, 8,000; zinc, 20,000; iron, 28,000 mg; iodine, 200 mg; cobalt, 100 mg; selenium, 20 mg; antioxidants, 300 mg.

ME in all diets was calculated to be around 2,975 kcal/kg, and Ca ranged from 0.95% to 0.97%.

1) The values of each parameter represent the mean values±SD of triplicate analyses (in dry mater).

YBP, yam bean pulp; SD, standard deviation.

**Table 3:** Composition and nutrient level of the experimental diets for the *in vivo* experiment

Ingredients, % (as-fed basis)	Dietary groups			

	0% YBP	4% YBP	8% YBP	12% YBP
Maize	52.7	47.3	41.9	36.5
Soybean meal	41.2	41.5	41.8	42.1
YBP	0.0	4.0	8.0	12.0
Palm oil	1.6	2.6	3.7	4.8
DL-methionine	0.2	0.2	0.2	0.2
L-lysine	0.5	0.5	0.5	0.5
Vit-mineral premix	0.3	0.3	0.3	0.3
Dicalcium phosphate	2.1	2.1	2.2	2.2
Calcium carbonate	1.0	1.0	1.0	1.0
Salt	0.5	0.5	0.5	0.5
Analyzed value (%)^1)^				
Crude protein	21.1±1.01	20.7±0.36	21.7±0.94	21.2±0.75
Crude fiber	3.2±0.34	3.9±0.59	4.4±0.29	4.9±0.45

Vit-mineral premixes provided the following per kilogram of premix: vit A, 1,500,000 IU; vit D3, 300,000 IU; vit E, 2,400 IU; vit K, 300 mg; thiamin, 300 mg; riboflavin, 600 mg; pyridoxine, 300 mg; vit 12, 4 mg; pantothenic acid, 1,500 mg; folic acid, 60 mg; nicotinic acid, 2,000 mg; biotin, 10 mg; copper, 24,000 mg; manganese, 8,000; zinc, 20,000; iron, 28,000 mg; iodine, 200 mg; cobalt, 100 mg; selenium, 20 mg; antioxidants, 300 mg.

ME in all diets was calculated to be around 2,975 kcal/kg, and Ca ranged from 0.95% to 0.97%.

1) The values of each parameter represent the mean values±SD of triplicate analyses (in dry mater).

YBP, yam bean pulp; SD, standard deviation.

**Table 4:** Effects of inclusion of yam bean pulp on the digestive organ traits of broilers reared up to 21 days of age

Items	Dietary groups			

	0% YBP	4% YBP	8% YBP	12% YBP
Organ weight (g/100 live BW)				
Proventriculus	0.85±0.10	0.86±0.07	0.84±0.09	0.83±0.04
Gizzard	3.59±0.68	3.54±0.25	3.66±0.46	3.46±0.25
Liver+gallbladder	2.81±0.34	2.56±0.19	2.59±0.17	2.24±0.56
Duodenum	1.30±0.10	1.15±0.11	1.25±0.29	1.23±0.14
Jejunum	2.48±0.34	2.56±0.19	2.49±0.24	2.49±0.15
Ileum	0.58±0.06	0.61±0.09	0.55±0.05	0.60±0.13
Intestinal length (cm/100 g live BW)				
Duodenum	6.45±0.54	6.27±0.48	6.72±0.39	6.25±0.56
Jejunum	21.21±2.13	22.66±1.81	21.96±1.77	21.49±1.61
Ileum	6.49±0.54	6.67±0.61	5.89±0.96	5.70±0.65

The values represent the mean±standard deviation of 5 replicate pens per treatment with 3 birds per replicate.

YBP, yam bean pulp; live BW, live bodyweight.

**Table 5:** Effects of inclusion of yam bean pulp on the growth performance of broilers reared up to 21 days of age

Items	Dietary groups			

	0% YBP	4% YBP	8% YBP	12% YBP
FI (g/bird/d)	34.0±2.11	34.3±3.57	33.5±2.20	34.6±2.47
BW gain (g/bird)	329.8±35.59	347.5±27.08	341.7±14.42	371.0±21.86
ADG (g/bird/d)	15.7±1.69	16.5±1.29	16.3±0.69	17.7±1.04
FCR	2.18±0.19	2.07±0.12	2.06±0.14	1.96±0.12

The values represent the mean±standard deviation of 5 replicate pens per treatment with 10 birds per replicate.

YBP, yam bean pulp; FI, feed intake; BW gain, body weight gain; ADG, average daily gain; FCR, feed conversion ratio.

**Table 6:** Effects of inclusion of yam bean pulp on the serum lipid profiles of broilers reared up to 21 days of age

Dietary groups	Triglyceride (mg/dL)	Cholesterol (mg/dL)	LDL (mg/dL)
0% YBP	33.10±6.55	127.30±14.69	51.60±8.17
4% YBP	33.10±8.86	127.90±13.70	45.50±10.51
8% YBP	26.10±3.52	124.30±29.16	47.00±18.33
12% YBP	30.40±4.83	154.60±5.19	57.50±6.30

The values represent the mean±standard deviation of 5 replicate pens per treatment with 2 birds per replicate.

LDL, low-density lipoproteins; YBP, yam bean pulp.
